# 4-Hydr­oxy-6-[(4-hydr­oxy-1-oxo-1,2-dihydro­phthalazin-6-yl)carbon­yl]phthalazin-1(2*H*)-one

**DOI:** 10.1107/S1600536809046509

**Published:** 2009-11-11

**Authors:** Rui-Sha Zhou, Jiang-Feng Song

**Affiliations:** a Department of Chemistry, North University of China, Taiyuan, Shanxi 030051, People’s Republic of China

## Abstract

In the crystal structure of the title compound, C_17_H_10_N_4_O_5_, the mol­ecules lie on twofold axes (through the ketone bridge C and O atoms). The dihedral angle between the two phthalazine rings is 52.25 (1)°. In the crystal, inter­molecular N—H⋯O and O—H⋯O inter­actions link the mol­ecules.

## Related literature

For the acyl­ate reaction of polycarboxyl­ate with hydrazine hydrate, see: Benniston *et al.* (1999 ▶); Hu *et al.* (2004 ▶).
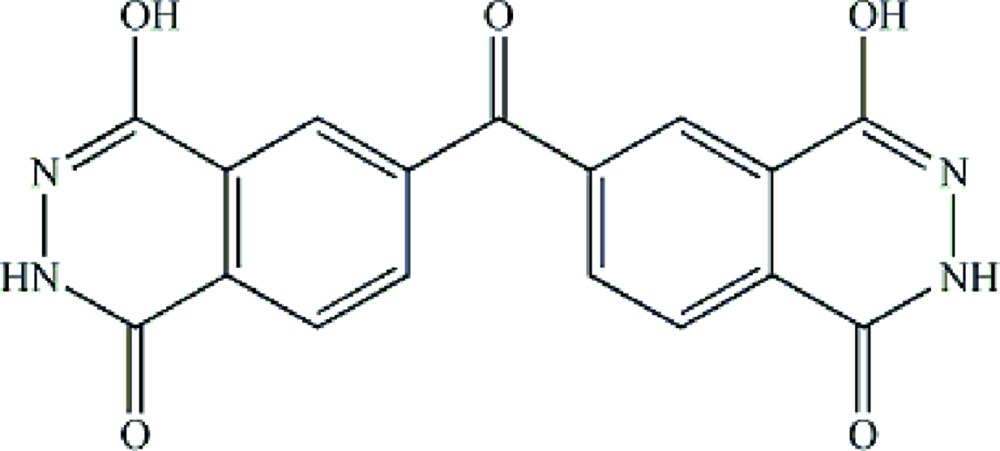


## Experimental

### 

#### Crystal data

C_17_H_10_N_4_O_5_
*M* *_r_* = 350.29
Monoclinic, 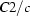

*a* = 11.576 (3) Å
*b* = 10.511 (3) Å
*c* = 12.274 (3) Å
β = 111.718 (4)°
*V* = 1387.4 (6) Å^3^
*Z* = 4
Mo *K*α radiation
μ = 0.13 mm^−1^
*T* = 293 K
0.30 × 0.25 × 0.10 mm

#### Data collection

Bruker SMART APEX CCD diffractometer
Absorption correction: multi-scan (*SADABS*; Sheldrick, 1995 ▶) *T*_min_ = 0.963, *T*_max_ = 0.987
3800 measured reflections
1370 independent reflections
774 reflections with *I* > 2σ(*I*)
*R*_int_ = 0.057

#### Refinement

*R*[*F* ^2^ > 2σ(*F* ^2^)] = 0.059
*wR*(*F* ^2^) = 0.161
*S* = 0.99
1370 reflections
120 parameters
H-atom parameters constrained
Δρ_max_ = 0.27 e Å^−3^
Δρ_min_ = −0.22 e Å^−3^

### 

Data collection: *SMART* (Bruker, 1999 ▶); cell refinement: *SAINT* (Bruker, 1999 ▶); data reduction: *SAINT*; program(s) used to solve structure: *SHELXS97* (Sheldrick, 2008 ▶); program(s) used to refine structure: *SHELXL97* (Sheldrick, 2008 ▶); molecular graphics: *SHELXTL* (Sheldrick, 2008 ▶); software used to prepare material for publication: *SHELXL97*.

## Supplementary Material

Crystal structure: contains datablocks I, global. DOI: 10.1107/S1600536809046509/jh2110sup1.cif

Structure factors: contains datablocks I. DOI: 10.1107/S1600536809046509/jh2110Isup2.hkl

Additional supplementary materials:  crystallographic information; 3D view; checkCIF report

## Figures and Tables

**Table 1:** Hydrogen-bond geometry (Å, °)

*D*—H⋯*A*	*D*—H	H⋯*A*	*D*⋯*A*	*D*—H⋯*A*
O1—H1⋯O2^i^	0.85	1.76	2.581 (3)	163
N2—H2⋯O1^ii^	0.86	2.19	3.034 (4)	168

Symmetry codes: (i) 
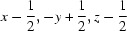
; (ii) 
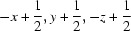
.

**Fig. 1.**
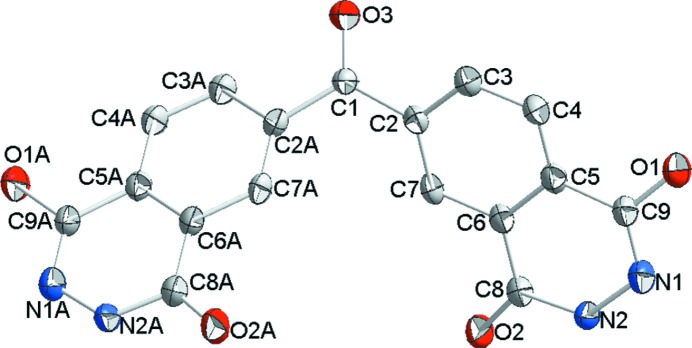
The molecular structure of the title compound shown with 30% probabilty ellipsoids [symmetry code: (A) 1 - x, y, 1.5 - z].

**Fig. 2.**
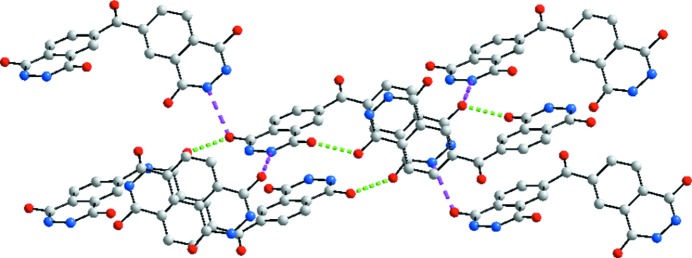
View of the crystal packing of the title compound, with O—H···O and N—H···O hydrogen bonds drawn as dashed lines [see Table 1 for details; H atoms not involved in hydrogen bonding have been omitted for clarity].

**Fig. 3.**
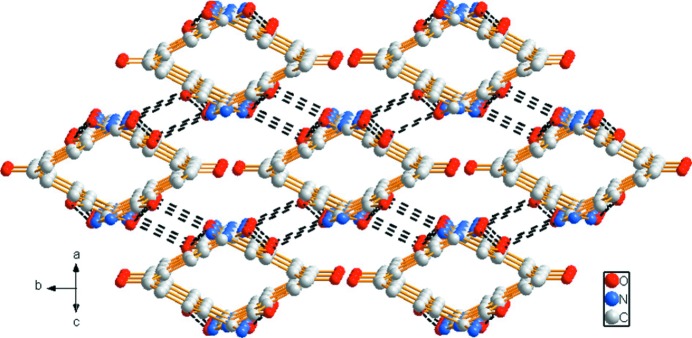
Three-dimensional network formed by hydrogen bonds (dashed lines).

## supplementary crystallographic information

### Comment

In situ hydrothermal acylate reaction of multidentate aromatic polycarboxylate with hydrazine hydrate has been investigated (Benniston *et al.*, 1999; Hu *et al.*, 2004). We intend to select 3, 3'-4, 4'-benzophenonetetracarboxylic dianhydride as the ligand to continue the exploration of the *in situ* acylate reaction. However, the crystals of the title compound were obtained unintentionally as the harvested product of the hydrothermal reaction of 3, 3'-4, 4'-benzophenonetetracarboxylic dianhydride, CoCl_2_ and hydrazine hydrate. In the title compound, a twofold axis lies in the C atom and O atom of the ketone bridge (Fig. 1). Each organic molecule connects six adjacent ones into a three-dimensional supramolecular network by intermolecular O—H···O (2.576 Å) and N—H···O (3.034 Å) hydrogen bonds (Fig. 2 and Fig. 3).

### Experimental

Yellow needle-like crystals of the title compound were synthesized hydrothermally from a mixture of CoCl_2_.H_2_O (0.0230 g), 3, 3'-4, 4'-benzophenonetetracarboxylic dianhydride (0.0641 g), hydrazine hydrate (0.028 ml), and deionized water (15 ml) in a 23 ml Teflon-lined stainless steel autoclave under autogenous pressure heated to 170 °C for 4 days and cooled to room temperature. Crystalline product was filtered, washed with distilled water, and dried at ambient temperature.

### Refinement

The H atoms were positioned geometrically and refined using riding model with C—H = 0.93 Å, N—H = 0.85 Å and isotropic displacement parameters *U*_iso_(H) = 1.2*U*(C_eq_ / N_eq_). However, the H of the O1 atom was located in a difference Fourier map refined as riding, with *U*_iso_(H) = 1.5*U*_eq_(O).

### Crystal data

**Table:**

C_17_H_10_N_4_O_5_	*F*(000) = 720
*M**_r_* = 350.29	*D*_x_ = 1.677 Mg m^−^^3^
Monoclinic, *C*2/*c*	Mo *K*α radiation, λ = 0.71073 Å
*a* = 11.576 (3) Å	θ = 2.7–26.0°
*b* = 10.511 (3) Å	µ = 0.13 mm^−^^1^
*c* = 12.274 (3) Å	*T* = 293 K
β = 111.718 (4)°	Needle-like, yellow
*V* = 1387.4 (6) Å^3^	0.30 × 0.25 × 0.10 mm
*Z* = 4	

### Data collection

**Table:**

Bruker SMART APEX CCD diffractometer	1370 independent reflections
Radiation source: fine-focus sealed tube	774 reflections with *I* > 2σ(*I*)
graphite	*R*_int_ = 0.057
ω scans	θ_max_ = 26.0°, θ_min_ = 2.7°
Absorption correction: multi-scan (*SADABS*; Sheldrick, 1995)	*h* = −14→12
*T*_min_ = 0.963, *T*_max_ = 0.987	*k* = −12→12
3800 measured reflections	*l* = −15→14

### Refinement

**Table:**

Refinement on *F*^2^	Primary atom site location: structure-invariant direct methods
Least-squares matrix: full	Secondary atom site location: difference Fourier map
*R*[*F*^2^ > 2σ(*F*^2^)] = 0.059	Hydrogen site location: inferred from neighbouring sites
*wR*(*F*^2^) = 0.161	H-atom parameters constrained
*S* = 0.99	*w* = 1/[σ^2^(*F*_o_^2^) + (0.0758*P*)^2^] where *P* = (*F*_o_^2^ + 2*F*_c_^2^)/3
1370 reflections	(Δ/σ)_max_ = 0.005
120 parameters	Δρ_max_ = 0.27 e Å^−^^3^
0 restraints	Δρ_min_ = −0.22 e Å^−^^3^

### Special details

**Table:**

Geometry. All e.s.d.'s (except the e.s.d. in the dihedral angle between two l.s. planes) are estimated using the full covariance matrix. The cell e.s.d.'s are taken into account individually in the estimation of e.s.d.'s in distances, angles and torsion angles; correlations between e.s.d.'s in cell parameters are only used when they are defined by crystal symmetry. An approximate (isotropic) treatment of cell e.s.d.'s is used for estimating e.s.d.'s involving l.s. planes.
Refinement. Refinement of *F*^2^ against ALL reflections. The weighted *R*-factor *wR* and goodness of fit *S* are based on *F*^2^, conventional *R*-factors *R* are based on *F*, with *F* set to zero for negative *F*^2^. The threshold expression of *F*^2^ > σ(*F*^2^) is used only for calculating *R*-factors(gt) *etc*. and is not relevant to the choice of reflections for refinement. *R*-factors based on *F*^2^ are statistically about twice as large as those based on *F*, and *R*- factors based on ALL data will be even larger.

### Fractional atomic coordinates and isotropic or equivalent isotropic displacement parameters (Å^2^)

**Table:**

	*x*	*y*	*z*	*U*_iso_*/*U*_eq_	
O3	0.5000	−0.2105 (3)	0.7500	0.0411 (9)	
O1	0.1272 (2)	0.0713 (2)	0.23363 (19)	0.0468 (7)	
H1	0.0902	0.1164	0.1732	0.070*	
O2	0.5078 (2)	0.3361 (2)	0.5277 (2)	0.0512 (8)	
N1	0.2410 (3)	0.2518 (3)	0.2824 (2)	0.0406 (8)	
N2	0.3389 (3)	0.3149 (3)	0.3641 (2)	0.0367 (8)	
H2	0.3513	0.3916	0.3467	0.044*	
C6	0.3861 (3)	0.1464 (3)	0.5017 (3)	0.0303 (8)	
C9	0.2158 (3)	0.1394 (3)	0.3114 (3)	0.0356 (9)	
C5	0.2831 (3)	0.0802 (3)	0.4238 (2)	0.0314 (8)	
C4	0.2480 (3)	−0.0367 (3)	0.4556 (3)	0.0362 (9)	
H4	0.1781	−0.0790	0.4052	0.043*	
C7	0.4564 (3)	0.0920 (3)	0.6103 (2)	0.0320 (8)	
H5	0.5254	0.1346	0.6620	0.038*	
C8	0.4182 (3)	0.2707 (3)	0.4689 (3)	0.0340 (8)	
C3	0.3181 (3)	−0.0884 (3)	0.5624 (3)	0.0363 (9)	
H3	0.2956	−0.1668	0.5835	0.044*	
C1	0.5000	−0.0954 (4)	0.7500	0.0281 (10)	
C2	0.4228 (3)	−0.0255 (3)	0.6404 (2)	0.0297 (8)	

### Atomic displacement parameters (Å^2^)

**Table:**

	*U*^11^	*U*^22^	*U*^33^	*U*^12^	*U*^13^	*U*^23^
O3	0.046 (2)	0.030 (2)	0.0369 (19)	0.000	0.0043 (17)	0.000
O1	0.0448 (16)	0.0439 (16)	0.0330 (14)	−0.0014 (13)	−0.0076 (12)	0.0032 (11)
O2	0.0573 (18)	0.0442 (16)	0.0328 (14)	−0.0200 (14)	−0.0057 (13)	0.0032 (12)
N1	0.0419 (18)	0.0406 (19)	0.0291 (15)	0.0021 (15)	0.0011 (14)	−0.0015 (13)
N2	0.0413 (18)	0.0273 (16)	0.0286 (15)	−0.0031 (13)	−0.0020 (14)	0.0025 (12)
C6	0.033 (2)	0.032 (2)	0.0226 (16)	−0.0007 (15)	0.0059 (16)	−0.0040 (14)
C9	0.038 (2)	0.033 (2)	0.0260 (18)	0.0018 (17)	0.0002 (17)	−0.0027 (15)
C5	0.0308 (19)	0.033 (2)	0.0254 (16)	0.0047 (16)	0.0051 (15)	−0.0033 (14)
C4	0.037 (2)	0.032 (2)	0.0297 (18)	−0.0059 (16)	0.0016 (17)	−0.0023 (15)
C7	0.0294 (19)	0.035 (2)	0.0231 (17)	−0.0043 (16)	−0.0001 (15)	−0.0035 (14)
C8	0.038 (2)	0.033 (2)	0.0254 (17)	−0.0009 (17)	0.0053 (16)	−0.0021 (15)
C3	0.039 (2)	0.033 (2)	0.0302 (18)	−0.0042 (17)	0.0050 (16)	−0.0010 (16)
C1	0.028 (3)	0.024 (3)	0.027 (2)	0.000	0.005 (2)	0.000
C2	0.035 (2)	0.030 (2)	0.0217 (17)	0.0007 (15)	0.0074 (16)	−0.0046 (13)

### Geometric parameters (Å, °)

**Table:**

O3—C1	1.210 (5)	C9—C5	1.450 (4)
O1—C9	1.323 (4)	C5—C4	1.394 (4)
O1—H1	0.8501	C4—C3	1.373 (4)
O2—C8	1.231 (4)	C4—H4	0.9300
N1—C9	1.298 (4)	C7—C2	1.386 (4)
N1—N2	1.374 (4)	C7—H5	0.9300
N2—C8	1.357 (4)	C3—C2	1.401 (4)
N2—H2	0.8600	C3—H3	0.9300
C6—C7	1.401 (4)	C1—C2^i^	1.502 (4)
C6—C5	1.406 (4)	C1—C2	1.502 (4)
C6—C8	1.455 (5)		
			
C9—O1—H1	109.5	C5—C4—H4	120.5
C9—N1—N2	116.6 (3)	C2—C7—C6	119.7 (3)
C8—N2—N1	127.8 (3)	C2—C7—H5	120.1
C8—N2—H2	116.1	C6—C7—H5	120.1
N1—N2—H2	116.1	O2—C8—N2	119.5 (3)
C7—C6—C5	119.5 (3)	O2—C8—C6	125.8 (3)
C7—C6—C8	120.8 (3)	N2—C8—C6	114.6 (3)
C5—C6—C8	119.7 (3)	C4—C3—C2	121.5 (3)
N1—C9—O1	119.2 (3)	C4—C3—H3	119.3
N1—C9—C5	123.8 (3)	C2—C3—H3	119.3
O1—C9—C5	117.0 (3)	O3—C1—C2^i^	119.3 (2)
C4—C5—C6	120.5 (3)	O3—C1—C2	119.3 (2)
C4—C5—C9	122.3 (3)	C2^i^—C1—C2	121.5 (4)
C6—C5—C9	117.2 (3)	C7—C2—C3	119.7 (3)
C3—C4—C5	119.0 (3)	C7—C2—C1	122.8 (3)
C3—C4—H4	120.5	C3—C2—C1	117.3 (3)
			
C9—N1—N2—C8	4.9 (5)	N1—N2—C8—O2	173.9 (3)
N2—N1—C9—O1	−176.2 (3)	N1—N2—C8—C6	−6.8 (5)
N2—N1—C9—C5	1.1 (5)	C7—C6—C8—O2	2.4 (5)
C7—C6—C5—C4	2.4 (5)	C5—C6—C8—O2	−177.7 (3)
C8—C6—C5—C4	−177.5 (3)	C7—C6—C8—N2	−176.8 (3)
C7—C6—C5—C9	−178.3 (3)	C5—C6—C8—N2	3.1 (4)
C8—C6—C5—C9	1.8 (5)	C5—C4—C3—C2	0.8 (5)
N1—C9—C5—C4	175.2 (3)	C6—C7—C2—C3	−0.6 (5)
O1—C9—C5—C4	−7.5 (5)	C6—C7—C2—C1	174.2 (3)
N1—C9—C5—C6	−4.1 (5)	C4—C3—C2—C7	0.6 (5)
O1—C9—C5—C6	173.2 (3)	C4—C3—C2—C1	−174.4 (3)
C6—C5—C4—C3	−2.4 (5)	O3—C1—C2—C7	−148.7 (2)
C9—C5—C4—C3	178.4 (3)	C2^i^—C1—C2—C7	31.3 (2)
C5—C6—C7—C2	−0.9 (5)	O3—C1—C2—C3	26.2 (3)
C8—C6—C7—C2	179.0 (3)	C2^i^—C1—C2—C3	−153.8 (3)

Symmetry codes: (i) −*x*+1, *y*, −*z*+3/2.

### Hydrogen-bond geometry (Å, °)

**Table:**

*D*—H···*A*	*D*—H	H···*A*	*D*···*A*	*D*—H···*A*
O1—H1···O2^ii^	0.85	1.76	2.581 (3)	163
N2—H2···O1^iii^	0.86	2.19	3.034 (4)	168

Symmetry codes: (ii) *x*−1/2, −*y*+1/2, *z*−1/2; (iii) −*x*+1/2, *y*+1/2, −*z*+1/2.
